# “I assume that my doctor will then guide me”: Attitudes and Expectations in the Context of Dementia Risk Estimation

**DOI:** 10.1002/alz.092186

**Published:** 2025-01-09

**Authors:** Constanze Hübner, Annika Baumeister, Michelle Gerards, Yahveth Cantero‐Fortiz, Federica Ribaldi, Julia Braun, Mercè Boada, Giovanni B. Frisoni, Frank Jessen, Ayda Rostamzadeh, Carolin Schwegler, Björn Schmitz‐Luhn, Christiane Woopen

**Affiliations:** ^1^ Center for Life Ethics, University of Bonn, Bonn Germany; ^2^ Department of Psychiatry and Psychotherapy, Medical Faculty, University of Cologne, Cologne Germany; ^3^ Ace Alzheimer Center Barcelona – International University of Catalunya (UIC), Barcelona Spain; ^4^ Geneva Memory Center, Department of Rehabilitation and Geriatrics, Geneva University Hospitals, Geneva Switzerland; ^5^ Laboratory of Neuroimaging of Aging, University of Geneva, Geneve Switzerland; ^6^ Network Center for Biomedical Research in Neurodegenerative Diseases (CIBERNED), Madrid Spain; ^7^ Laboratory of Neuroimaging of Aging, University of Geneva, Geneva Switzerland; ^8^ German Center for Neurodegenerative Diseases (DZNE), Bonn Germany; ^9^ Excellence Cluster on Cellular Stress Responses in Aging‐Associated Diseases (CECAD), University of Cologne, Cologne Germany; ^10^ Faculty of Arts and Humanities, University of Cologne, Cologne Germany

## Abstract

**Background:**

Driven by (bio‐)medical and technical developments, advanced non‐invasive methods for estimating the risk of Alzheimer’s dementia (ADD) are increasingly emerging. In the future, such methods could eventually become available for individuals in asymptomatic and preclinical stages of Alzheimer’s disease (e.g. individuals with subjective cognitive decline (SCD)). Disclosure of individual ADD risk can bring new challenges for those affected, which can be linked to ethically relevant aspects such as identity and self‐perception. Such considerations can become more and more relevant in the context of ADD risk estimation in clinical care. In a qualitative approach, the ethical subproject of the PreTAD project (The Predictive Turn in Alzheimer’s Disease: Ethical, Clinical, Linguistic and Legal Aspects) aims, among other objectives, to investigate the attitudes and expectations of persons with various experiences with ADD regarding ADD risk estimation.

**Method:**

In the qualitative part of the study, n = 30 interviews out of N = 40 are intended to be conducted with first‐degree relatives of persons with ADD and patients with SCD diagnosed ≤4 weeks or >4 weeks ago. The interviews address inter alia attitudes, individual experiences, and expectations regarding ADD risk estimation. Thematic analysis (Kuckartz & Rädiker 2023) is employed to analyze interview transcripts.

**Result:**

Preliminary analysis of n = 23 interviews revealed a spectrum of attitudes and expectations regarding ADD risk estimation shaped by individual experiences with ADD, care experience, or the healthcare system. Across groups, the majority expressed a positive attitude toward ADD risk estimation. The hypothetical offer of ADD risk estimation was frequently perceived as a way of gaining control by learning about one’s risk status. Hopes regarding potential risk knowledge included the anticipated opportunity to proactively engage in one’s health under medical guidance and the chance to initiate self‐determined advance care measures, which was emphasized by individuals with care experience. Potential psychological strain due to risk knowledge was rather mentioned by individuals with SCD.

**Conclusion:**

Preliminary findings highlighted diverse expectations associated with ADD risk knowledge touching upon ethically relevant aspects such as (self‐)responsibility and self‐determination. Our findings can serve as valuable starting points for an ethically guided approach to handling ADD risk estimation in clinical practice.

